# Retracted: Sagunja-Tang Improves Lipid Related Disease in a Postmenopausal Rat Model and HepG2 Cells

**DOI:** 10.1155/2016/9676590

**Published:** 2016-03-31

**Authors:** Evidence-Based Complementary and Alternative Medicine

The article titled “Sagunja-Tang Improves Lipid Related Disease in a Postmenopausal Rat Model and HepG2 Cells” [1] has been retracted at the request of the authors as it was found to be similar to the following previously published article: “Palmiwon attenuates hepatic lipid accumulation and hyperlipidemia in a menopausal rat model,” by Hiroe Go, Jin Ah Ryuk, Hye Won Lee, and Byoung Seob Ko, in Menopause: The Journal of The North American Menopause Society: 2015, Volume 22, Issue 8, pp. 872–884.
